# Population pharmacokinetic analysis of pirtobrutinib, a non-covalent BTK inhibitor, in patients with hematological malignancies from the Phase 1/2 BRUIN study

**DOI:** 10.1007/s00280-026-04951-4

**Published:** 2026-09-24

**Authors:** Robert Bell, Lisa M O’Brien, Eunice Yuen, Dan Liu, Sonya C. Chapman

**Affiliations:** Eli Lilly and Company, Indianapolis, IN USA

**Keywords:** Pirtobrutinib, Pharmacokinetics, Hematological malignancies

## Abstract

**Purpose:**

This study aimed to characterize the pharmacokinetics (PK) of pirtobrutinib, a highly selective and noncovalent Bruton’s tyrosine kinase (BTK) inhibitor, in adult patients with hematological malignancies using data from the Phase 1/2 BRUIN study (NCT03740529).

**Methods:**

Pirtobrutinib was administered as monotherapy at doses ranging from 25 to 300 mg once daily (QD). PK samples were collected at multiple time points, including intensive sampling on Cycle (C) 1 Day (D) 1, C1D8, C2D1, and C4D1 for Phase 1 dose-escalation, and sparse sampling on C1D8 and C4D1 for Phase 1 dose-expansion and Phase 2. Population PK (popPK) analysis used nonlinear mixed-effects modeling.

**Results:**

Overall, 4487 evaluable pirtobrutinib concentrations obtained from 595 patients were included in the popPK analysis. Pirtobrutinib PK was well characterized by a 2-compartment model with linear clearance and 4-transit compartments for absorption. The mean elimination half-life was estimated to be 18.8 h. At the recommended Phase 2 dose (200 mg QD), the steady-state mean maximum drug concentration was 6460ng/mL, the mean minimum was 2260ng/mL, and the mean area under the concentration-time curve was 91300ng*h/mL. Pirtobrutinib disposition was significantly affected by body weight, renal function, and serum albumin, but not by formulation, cancer type, age, sex, race, ethnicity, or mild hepatic impairment.

**Conclusion:**

With the proposed starting dose of 200 mg QD, most patients (96%) were expected to exceed 90% BTK inhibition, across the entire dosing interval, indicating extensive and durable engagement of the drug target. No dose adjustments based on body weight, serum albumin or renal function were recommended.

**Clinical trial registration:**

NCT03740529.

**Supplementary Information:**

The online version contains supplementary material available at https://doi.org/10.1007/s00280-026-04951-4.

## Introduction

Hematological malignancies, such as chronic lymphocytic leukemia (CLL), small lymphocytic lymphoma (SLL), and mantle cell lymphoma (MCL) are types of B cell malignancies characterized by the increased production of mature but dysfunctional B lymphocytes [1, 2]. Bruton’s tyrosine kinase (BTK), a non-receptor enzyme, plays a critical role in B cell receptor signaling and is essential for the proliferation and survival of B cells. Irreversible BTK inhibitors have demonstrated efficacy in treating B cell malignancies, however, their use is often limited by off-target effects and the development of acquired resistance [3, 4].

Pirtobrutinib is a novel, highly selective, reversible, noncovalent BTK inhibitor designed to overcome the limitations of current irreversible BTK inhibitors. In January 2023, Food and Drug Administration (FDA) granted accelerated approval to pirtobrutinib for the treatment of adult patients with relapsed or refractory MCL after 2 lines of systemic therapy, including a BTK inhibitor. Later that year, pirtobrutinib was also approved for the treatment of adults with CLL/SLL who have received at least 2 prior lines of therapy [5, 6]. Pirtobrutinib monotherapy has demonstrated sustained efficacy and a favorable safety profile across multiple clinical settings, including post-cBTKi, cBTKi-naïve relapsed/refractory, and treatment-naïve disease [7–9] supporting global regulatory approvals for the treatment of adult patients with relapsed/refractory CLL previously treated with a cBTKi.

To ensure that patients are sufficiently exposed to pirtobrutinib for an adequate duration during the dosing interval, it is crucial to characterize its pharmacokinetics (PK). This characterization helps in achieving extensive time on target, which is essential for the drug’s efficacy. Additionally, individualizing the dose based on patient characteristics, such as body weight, renal function, and serum albumin levels, which may affect PK exposures, is necessary to optimize therapeutic outcomes and minimize adverse effects.

The present study aimed to characterize the PK of pirtobrutinib in patients with hematological malignancies including patients with MCL, relapsed or refractory CLL/SLL and B cell non-Hodgkin’s lymphoma (NHL). Additionally, the study evaluated the impact of intrinsic factors (such as age, sex, race/ethnicity, body weight, cancer type, and renal and hepatic function) and extrinsic factors (such as formulation) on pirtobrutinib disposition in this patient population.

## Methods

### Study design and patients

Details related to the BRUIN trial (NCT03740529) study design and patient population have been published previously [10, 11]. Briefly, the BRUIN trial is an open-label, multi-center study assessing the safety and efficacy of oral pirtobrutinib in patients with MCL, CLL/SLL, and NHL who have either failed standard treatment or experienced intolerance to it.

The study involved a Phase 1 dose escalation followed by a dose expansion and Phase 2 cohorts. In the Phase 1 dose-escalation, pirtobrutinib was given at 7 different doses, ranging from 25 mg to 300 mg once daily (QD): specifically, 25 mg, 50 mg, 100 mg, 150 mg, 200 mg, 250 mg, and 300 mg. By the end of this Phase, the recommended Phase 2 dose (RP2D) was established at 200 mg QD. Patients in both the Phase 1 expansion and Phase 2 cohorts received the RP2D. They continued to receive pirtobrutinib as monotherapy until they experienced disease progression, unacceptable toxicity, or another reason for treatment discontinuation. Pirtobrutinib was administered as the initial tablet formulation (T1) earlier in the study and as the commercial tablet formulation (T2) later in the study with 210 patients (35%) initially received T1 and 385 (65%) received T2.

### Pharmacokinetic sampling

Intense PK sample collection was scheduled on Cycle 1 Day 1, Cycle 1 Day 8, Cycle 2 Day 1, and Cycle 4 Day 1. Cycles were 28 days in duration. On these days, samples were collected predose (within 1 h prior to dosing), and postdose at 1, 2, and 4 h (± 15 min) and 8 h (± 30 min). For Phase 1 dose-expansion and Phase 2, sparse PK sample collection was scheduled on Cycle 1 Day 8 and Cycle 4 Day 1. On these days, a single predose PK sample was collected.

### Analytical method

Plasma samples were analyzed using validated liquid-liquid extraction followed by High-Performance Liquid Chromatography coupled with Tandem Mass Spectrometry (HPLC-MS/MS) detection method. Initially, the method was validated with a lower limit of quantitation (LLOQ) of 1 ng/mL and an upper limit of quantitation (ULOQ) of 1000 ng/mL. Subsequently, the method was revalidated, achieving an LLOQ of 20 ng/mL and a ULOQ of 20,000 ng/mL. Samples exceeding the ULOQ were diluted and reanalyzed to ensure results fell within the calibrated ranges. The inter-assay precision and accuracy values were within the acceptable range. Cross-validation confirmed that similar results were obtained using both methods. The analysis used an interim data cutoff of 31 January 2022.

### Population pharmacokinetic modelling

The popPK analysis was conducted using nonlinear mixed-effects modeling techniques, implemented in NONMEM version 7.4.2 (ICON Development Systems, Gaithersburg, MD, USA) and Perl Speaks NONMEM ^®^ (PsN) version 4.8.1 (2018–2019 by Mats Karlsson, Rikard Nordgren, Svetlana Freiberga, Sebastian Ueckert, and Gunnar Yngman). First-order conditional estimation with interaction was used as the estimation method. Graphical data visualization, evaluation of NONMEM output, construction of goodness-of-fit plots, and simulations were conducted using R version 4.1.2, R Foundation for Statistical Computing, Vienna, Austria). Elimination half-life was calculated from individual post hoc parameter estimates as

$$\:{t}_{1/2}=\frac{\mathrm{ln}\left(2\right)}{\beta\:}\:\mathrm{a}\mathrm{n}\mathrm{d}\:\beta\:\:=\frac{\left[\left(k12\:+\:k21\:+\:k10\right)-\:\sqrt{{\left(k12\:+\:k21\:+\:k10\right)}^{2}-\:4\cdot k21 \cdot k10}\right]}{2},$$

where, $$\:k10\:=\frac{CL}{Vc}$$, $$\:k12\:=\frac{Q}{Vc}$$, and $$\:k21\:=\frac{Q}{Vp}$$.

#### Base model development

A series of 1-, 2-, and 3-compartment PK models were evaluated to identify the model which best described the pirtobrutinib concentration data in terms of apparent total body clearance following oral administration (CL/F), central volume (Vc/F), peripheral volume (Vp/F), and intercompartmental clearance (Q/F). Interpatient variability was assumed to be log-normally distributed, and variability terms were investigated for all PK model parameters. Covariance between parameters was assessed using an omega block. Proportional, additive, and combined additive/proportional residual error models were evaluated. Once inter-patient variability and residual error models were determined, inter-occasion variability was assessed on the PK parameters. Key criteria for model selection were (1) convergence of the estimation and covariance routines; (2) reasonable parameter and variance estimates based on the known PK of the compound; (3) acceptable precision of the parameter and variance estimates; and (4) graphical evaluation using prediction- or simulation-based metrics to confirm that the model accurately characterizes the data and shows no obvious misspecification.

#### Final model development

Once a structural and statistical base model was established, potentially significant patient factors were evaluated for their influence on the disposition of pirtobrutinib. Patient factors included in the assessment are provided in Table 1.

**Table 1:** Potentially clinically relevant intrinsic or extrinsic factors

Covariate	Type	Parameters Tested
Age	Continuous	MTT, CL/F, V/F
Sex	Categorical	MTT, CL/F, V/F
Race	Categorical	CL/F
Ethnicity	Categorical	CL/F
Body weight	Continuous	CL/F, V/F
Albumin	Continuous	CL/F, V/F
Renal function^a^	Continuous	CL/F
Hepatic function^b^	Categorical	CL/F
T1/T2 formulation	Categorical	F, MTT
Cancer type	Categorical	MTT, CL/F, V/F

AST = aspartate aminotransferase; CL/F = apparent total body clearance of drug calculated after extra-vascular administration; CYP = cytochrome P450; F = bioavailability of drug; eGFR = estimated glomerular filtration rate; MTT = mean transit time; TBI = total bilirubin; ULN = upper limit of normal; V/F = apparent volume of distribution

^a^Assessed using eGFR. eGFR = 170 * [serum creatinine (mg/dL)]^-0.999 * [age]^-0.176 * [0.762 if patient is female] * [1.18 if patient is black] * [serum urea nitrogen (mg/dL)]^-0.17 * [serum albumin (g/dL)]^0.318

^b^As determined by the National Cancer Institute Organ Dysfunction Working Group (NCI-ODWG) criteria for hepatic dysfunction [17]. Classified as normal (TBI ≤ ULN and AST ≤ ULN), mild impairment (TBI ≤ 1.5 * ULN and AST > ULN) or (TBI ≤ 1.5 · ULN and TBI > ULN), or moderate impairment (1.5 · ULN < TBI ≤ 3 · ULN) and severe impairment (TBI > 3 · ULN)

An assessment of covariate effects was conducted. Criteria for inclusion in a full model was based on.

- Convergence of the estimation and covariance routines with good precision of the parameter and variance estimates.
- Reasonable parameter and variance estimates based upon the known pharmacokinetics.
- Statistically significant difference in the minimum objective function (MOF) value for forward inclusion (≥ 6.635 points for 1 degree of freedom, *p* < 0.01, based on χ^2^ distribution),
- A decrease in the relative interpatient variability estimate for the PK parameter on which it was tested by at least 10%,
- Clinical relevance change in the PK parameter estimates by at least 15% for categorical covariates or 20% at the 5th or 95th percentile of continuous covariate range .
- Agreement between predicted and observed concentrations, as assessed by visual inspection.

Covariates retained in the final model were those resulting in a significant increase in MOF (≥ 10.828 points for 1 degree of freedom, *p* < 0.001, based on χ^2^ distribution) using backward elimination.

Continuous covariates were tested for relationships with relevant PK parameters using linear, exponential, and power models. Categorical covariates were tested using a proportional model. Model development identified a significant relationship between body weight and pirtobrutinib exposure. Per the study protocol, strong and moderate CYP3A4 inhibitors and inducers were prohibited concomitant medications. Concomitant CYP3A modulation was therefore not evaluated as a covariate in the population PK model.

#### Model evaluation

A bootstrap analysis was performed to assess the precision of the final parameter estimates of the model. The analysis was conducted using the bootstrap routine in PsN, with a total of 1000 bootstrap replicates. The 95% confidence intervals (CIs) for each parameter were calculated using the 2.5th and 97.5th percentile values from the distribution of bootstrap parameter values. Bootstrap resampling was stratified by study phase to ensure an appropriate proportion of data from serial and sparse sampling, maintaining the composition of the bootstrap datasets similar to the final analysis dataset.

A VPC was performed to ensure the model’s fidelity with the observed data. VPC simulations were conducted in NONMEM and summarized/plotted in R. The 90% prediction intervals of the profiles were calculated using the 5th and 95th percentiles of 200 simulations. Prediction-correction of VPCs was applied as previously described [12].

### Handling of missing data

Missing data and outliers were handled according to the FDA Guidance for Industry [13]. Data visualization and statistical analysis techniques were used to assess the potential impact of missing data and outliers on the analyses.

The datasets were inspected to identify missing data. Records in the NONMEM dataset containing missing dates or times essential to PK/PD analysis were excluded from the analysis. These records were identified as having doses with missing date or time; samples with missing date or time; samples without an associated dose, and safety events or efficacy outcomes with missing date. Missing or implausible values of independent variables (patient characteristic data) were replaced with the median value for the patient population. Of 4867 concentrations from 611 patients, records with missing or inadequate dosing information (*n* = 88) or sample date/time (*n* = 60), samples below the limit of quantification (*n* = 213 pre-dose; *n* = 17 on-treatment; <1% of records), and 2 implausible concentrations were excluded, yielding 4487 evaluable concentrations from 595 patients.

## Results

### Patient characteristics

Baseline demographics and clinical characteristics of patients included in the pirtobrutinib population PK analysis (*n* = 595) are presented in Table 2. Patients had a median age of 68.0 years (range: 27–95 years) and a median body weight of 77 kg (range: 36–153 kg). There were 394 male participants (66%) and 201 female participants (34%). At baseline, 18% of patients had mild hepatic impairment and 51% had mild renal impairment.

**Table 2:** Baseline demographics and characteristics

Parameters	Population PK *n* = 595
Median age, years (range)	68 (27–95)
Sex, n (%)	
Female	201 (34)
Male	394 (66)
Race, n (%)	
White	509 (86)
Black or African American	17 (3)
Asian	39 (7)
Other^a^	29 (5)
Not reported	1 (< 1)
Ethnic origin, n (%)	
Non-Hispanic	548 (92)
Hispanic	23 (4)
Not reported	24 (4)
Median weight, kg (range)^b^	77 (36–153)
Median BMI, kg/m^2^ (range)^c^	26 (14–47)
Median serum albumin, g/L (range)	41 (19–57)
Median eGFR, mL/min/1.73 m^2^ (range)^d^	72 (22–132)
Renal function (n, %)^e^	
Normal	121 (20)
Mild impairment	304 (51)
Moderate impairment	166 (28)
Severe impairment	4 (1)
Hepatic function, n, (%)^f^	
Normal	474 (80)
Mild impairment	106 (18)
Moderate impairment	13 (2)
Severe impairment	1 (< 1)
Not reported	1 (< 1)
Cancer type, n (%)	
MCL	140 (23)
CLL/SLL	263 (44)
All other NHL patients	192 (32)
Formulation of first dose, n (%)	
T1	210 (35)
T2	385 (65)

AST = aspartate aminotransferase; CLL = chronic lymphocytic leukemia; %CV = percent coefficient of variation; eGFR = estimated glomerular filtration rate; MCL = mantle cell lymphoma; MDRD = Modification of Diet in Renal Disease; N = number of patients; PK = pharmacokinetics; SLL = small lymphocytic lymphoma; TBI = total bilirubin; ULN = upper limit of normal

^a^Includes American Indian or Alaska native, Native Hawaiian or other Pacific Islander, or Other

^b^Baseline body weight missing for 2 patients (*n* = 593)

^c^Height missing for 31 participants (*n* = 564)

^d^eGFR as calculated by the Modification of Diet in Renal Disease Study Group equation (MDRD-6) = 170 · [serum creatinine (mg/dL)]^-0.999 · [age]^-0.176 · [serum urea nitrogen (mg/dL)]^-0.17 · [serum albumin (g/dL)]^0.318 · [0.762 if patient is female] · [1.18 if patient is black] [18]

^e^Classified as normal (eGFR ≥ 90 mL/minute/1.73 m^2^), mild impairment (60 mL/minute/1.73 m^2^ ≤ eGFR < 90 mL/minute/1.73 m^2^), moderate impairment (30 mL/minute/1.73 m^2^ ≤ eGFR < 60 mL/minute/1.73 m^2^), and severe impairment (15 mL/minute/1.73 m^2^ ≤ eGFR < 30 mL/minute/1.73 m^2^)

^f^As determined by the National Cancer Institute Organ Dysfunction Working Group (NCI-ODWG) criteria for hepatic dysfunction [17]. Classified as normal (TBI ≤ ULN and AST ≤ ULN), mild impairment (TBI ≤ 1.5 · ULN and AST > ULN) or (ULN < TBI ≤ 1.5 · ULN), moderate impairment (1.5 · ULN < TBI ≤ 3 · ULN), or severe impairment (TBI > 3 · ULN)

### Model

#### Model evaluation

The final popPK model included data from 595 patients, with 4487 pirtobrutinib concentrations. Pirtobrutinib PK data were best described by a linear 2-compartment structural model parameterized in terms of apparent CL/F, Vc/F, Vp/F, and Q/F with 4-transit compartments for absorption. Exponential interpatient variability terms were included for CL/F and mean transit time (MTT). Residual variability was accounted for by a proportional error structure. Model development identified a significant relationship between body weight and pirtobrutinib exposure. Body weight met the predefined criterion during individual covariate screening in the stepwise covariate modelling (SCM) process (*p* < 0.01). It was incorporated using allometric relationships with estimated exponents for CL/F, Q/F, Vc/F, and Vp/F (Table 3), added to the full model, and retained in the final model based on the predefined retention criterion (*p* < 0.001). Model parameter estimation was performed with overall high precision, and interindividual variability was generally low to moderate.

**Table 3:** Pharmacokinetic and covariate parameters in population model

Parameter	Parameter estimates (% SEE) Final Model	Final model bootstrap 95% CI
Bioavailability (F, fraction, Θ1)	1 fixed	1 fixed
MTT (h, Θ2)	1.08 (2.97)	(1.02, 1.14)
Clearance (CL, L/h, Θ3)	2.02 (1.66)	(1.96, 2.10)
Intercompartmental clearance (Q, L/h, Θ5)	8.38 (10.5)	(6.60, 10.7)
Central volume of distribution (Vc, L, Θ4)	32.8 (3.60)	(30.0, 35.4)
Peripheral volume of distribution (Vp, L, Θ6)	19.5 (5.49)	(17.4, 22.2)
Covariate Effects		
*Allometry on CL and Q*		
Body Weight (kg; Θ9)^a^	0.524 (11.5)	(0.386, 0.653)
*Allometry on Vc and Vp*		
Body Weight (kg; Θ10)^b^	0.785 (6.31)	(0.685, 0.881)
*Covariate effects on CL*		
eGFR (mL/min/1.73 m^2^; Θ12)^c^	0.00329 (31.0)	(0.00118, 0.00525)
Albumin (g/L; Θ11)^d^	-0.677 (16.7)	(-0.915, -0.457)
*Covariate effect on Vc*		
Albumin (g/L; Θ13)^e^	-0.513 (22.0)	(-0.759, -0.259)
Interindividual variability CV%		
MTT (Ω_2_)	25.0% (30.7)	(17.4, 33.1%)
CL (Ω_3_)	37.9% (7.61)	(34.8, 41.0%)
Interoccasion variability CV%		
MTT	45.9% (13.4)	(39.1, 52.7%)
Residual variability		
Proportional	0.205 (2.37)	(0.195, 0.214)

ALB = serum albumin; CI = confidence interval; CL = clearance; CV = coefficient of variation; F = relative bioavailability; eGFR = estimated glomerular filtration rate; MTT = mean transit time; NA = not applicable; Q = intercompartmental clearance; %SEE = percent standard error of the estimate; Vc = central volume of distribution; Vp = peripheral volume of distribution; WT=body weight at entry

^a^CL/F = Population estimate of CL*((WT/70)**Θ9); Q/F = Population estimate of Q*(WT/70)** Θ9)

^b^Vc/F = Population estimate of Vc*((WT/70)**Θ10); Vp/F = Population estimate of Vp*((WT/70)**Θ10)

^c^CL/F = Population estimate of CL*(exp(Θ12*(eGFR-74.96))

^d^CL/F = Population estimate of CL*((ALB/41.6)**Θ11)

^e^Vc/F = Population estimate of Vc* ((ALB/41.6)**Θ13)

In addition to body weight, serum albumin was identified as a statistically significant covariate on CL/F and Vc/F, and estimated glomerular filtration rate (eGFR) on CL/F. The inclusion of serum albumin and eGFR in the final model decreased the IIV on CL/F from 39.5% to 37.9%. Although the inclusion of these two covariates did not result in a relative decrease of ≥ 10% IIV., they were still retained in the final model based on a ≥ 20% change in PK parameter estimates between the 5th and 95th percentile values of these continuous covariates.

**Fig. 1:**
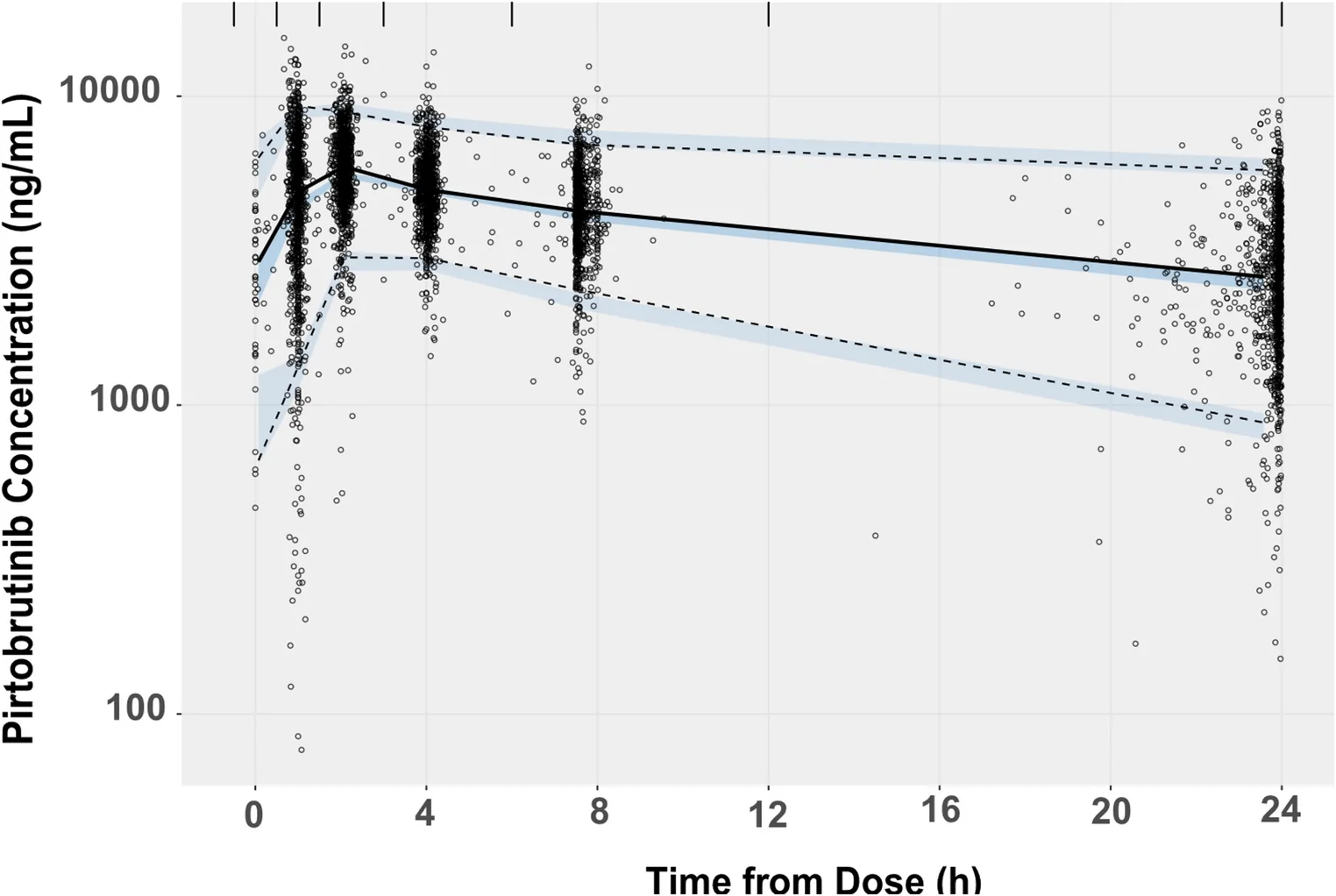
Prediction corrected visual predictive check of the population pharmacokinetic model for pirtobrutinib. NOTE: Solid black line represents median of observed data, dotted black lines represent the 5th and 95th percentiles of observed data. Shaded blue area depicts the model-predicted 95% confidence interval for 5th, 50th (median), and 95th percentiles

The 95% CIs of PK model parameters derived from bootstrap analysis showed adequate precision in parameter estimation and are included in Table 3. The pcVPC for the final popPK model showed good agreement between observed and model-predicted concentrations **(**Fig. 1).

### Impact of covariates on pirtobrutinib PK

Body weight, renal function (using eGFR), and serum albumin were identified as statistically significant covariates in the population PK model.

**Fig. 2:**
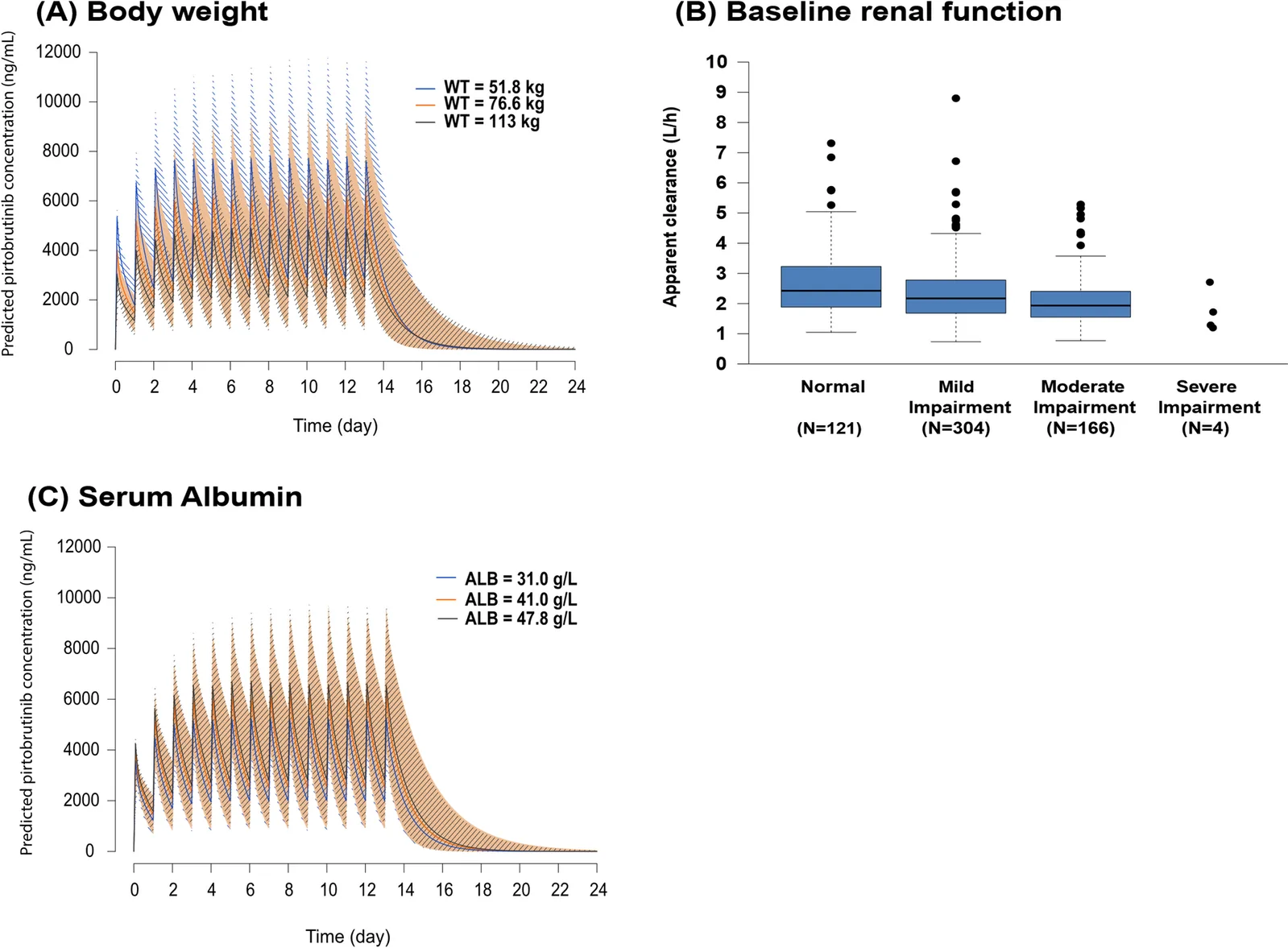
Impact of Various Factors on Pirtobrutinib Clearance-Simulated PK profiles at 200 mg QD. **(A)** PK profiles for patients with body weights at the 5th, 50th, and 95th percentiles. **(B)** Box and whisker plot for baseline renal function; box plot depict the 25th, 50th, and 75th percentiles. Whiskers represent 1.5 times the inter-quartile range. **(C)** PK profiles for patients with serum albumin values at 5th, 50th and 95th percentiles. NOTE: These simulations are based on the pirtobrutinib PK model, incorporating interindividual variability but not model uncertainty or residual error. The degree of overlap between these patient profiles illustrates the minimal impact of renal function on the PK profile when considering the natural PK variability in the patient population. Abbreviations: eGFR, estimated glomerular filtration rate; PK, pharmacokinetic; QD, once daily

To evaluate the clinical impact of body weight on the PK of pirtobrutinib, the PK of pirtobrutinib was simulated for a range of body weights (5th centile 51.8 kg, 50th centile 76.6 kg and 95th centile: 113 kg) based on those observed in the BRUIN Study (Fig. 2A). Simulation suggests the degree of overlap between these patient profiles illustrates the minimal impact body weight has on the PK profile when considering the degree of natural PK variability in the patient population.

The effect of renal function on the PK of pirtobrutinib was evaluated using eGFR as a continuous variable. There were 121 patients with normal renal function [eGFR ≥ 90 mL/min/1.73 m2], 304 patients with mild renal impairment [60 mL/min/1.73 m2 ≤ eGFR < 90 mL/min/1.73 m2], 166 patients with moderate renal impairment [30 mL/min/1.73 m2 ≤ eGFR < 60 mL/min/1.73 m2], and 4 patients with severe renal impairment [15 mL/min/1.73 m2 ≤ eGFR < 30 mL/min/1.73 m2]. To evaluate the effect of renal impairment, box plots illustrating the difference between apparent clearance grouped by renal function categorization are provided in Fig. 2B. This plot demonstrates the minimal change in apparent clearance between patients with normal renal function, and mild or moderate renal impairment. There is an insufficient number of patients in the severe renal impairment group to reliably estimate the change in CL/F in this population.

The effect of serum albumin on the PK of pirtobrutinib was also evaluated (Fig. 2C). Similarly, the PK of pirtobrutinib was simulated for a range of serum albumin values (5th centile 31.0 g/L, 50th centile 41.0 g/L and 95th centile: 47.8 g/L) based on those observed in the BRUIN Study. The degree of overlap between these patient profiles illustrates the minimal impact serum albumin has on the PK profile when considering the degree of natural PK variability in the patient population.

Additionally, the magnitude of the covariate effects on steady-state exposure for these three covariates is summarised in Figure S2. All covariate effects lie within, or only marginally outside, the 0.80–1.25 reference interval and are well within the interindividual variability in clearance (37.9% CV), supporting the conclusion that they are not clinically meaningful and do not warrant dose adjustment.

Age, sex, race, ethnicity, cancer type, mild hepatic impairment, and formulation did not significantly affect pirtobrutinib disposition (Figure S1). Notably, there were insufficient patient numbers to draw clear conclusions regarding the effect of moderate or severe hepatic impairment on the PK of pirtobrutinib (13 moderate [2%], 1 severe [< 1%]) (Table 2).

The mean elimination half-life of pirtobrutinib was estimated to be 18.8 h (37% CV). At the RP2D of 200 mg QD in Phase 2 patients, the model estimated geometric mean maximum observed drug concentration (C_max_) was 6460ng/mL, the mean minimum observed drug concentration (C_min_) was 2260ng/mL and the mean area under the concentration-time curve (AUC_0−24_) was 91300ng*h/mL.

### Predicted BTK inhibition

The extent of BTK inhibition in patients was evaluated by estimating the proportion of patients who achieve minimum steady-state concentrations that exceed the protein binding adjusted- IC_90_ for BTK in vitro (830 ng/mL) over the clinical dose range studied in the BRUIN study (25 mg to 300 mg QD). This IC90 value was derived from the in vitro IC50 (8.8 nM) for inhibition of BTK Y223 autophosphorylation in HEK293 cells stably expressing BTK [14] assuming Michaelis Menten kinetics (Emax = 100, Hill coefficient = 1) which was then adjusted for plasma protein binding (fu = 0.046 [16]). The simulation was based on 2000 patients.

**Fig. 3:**
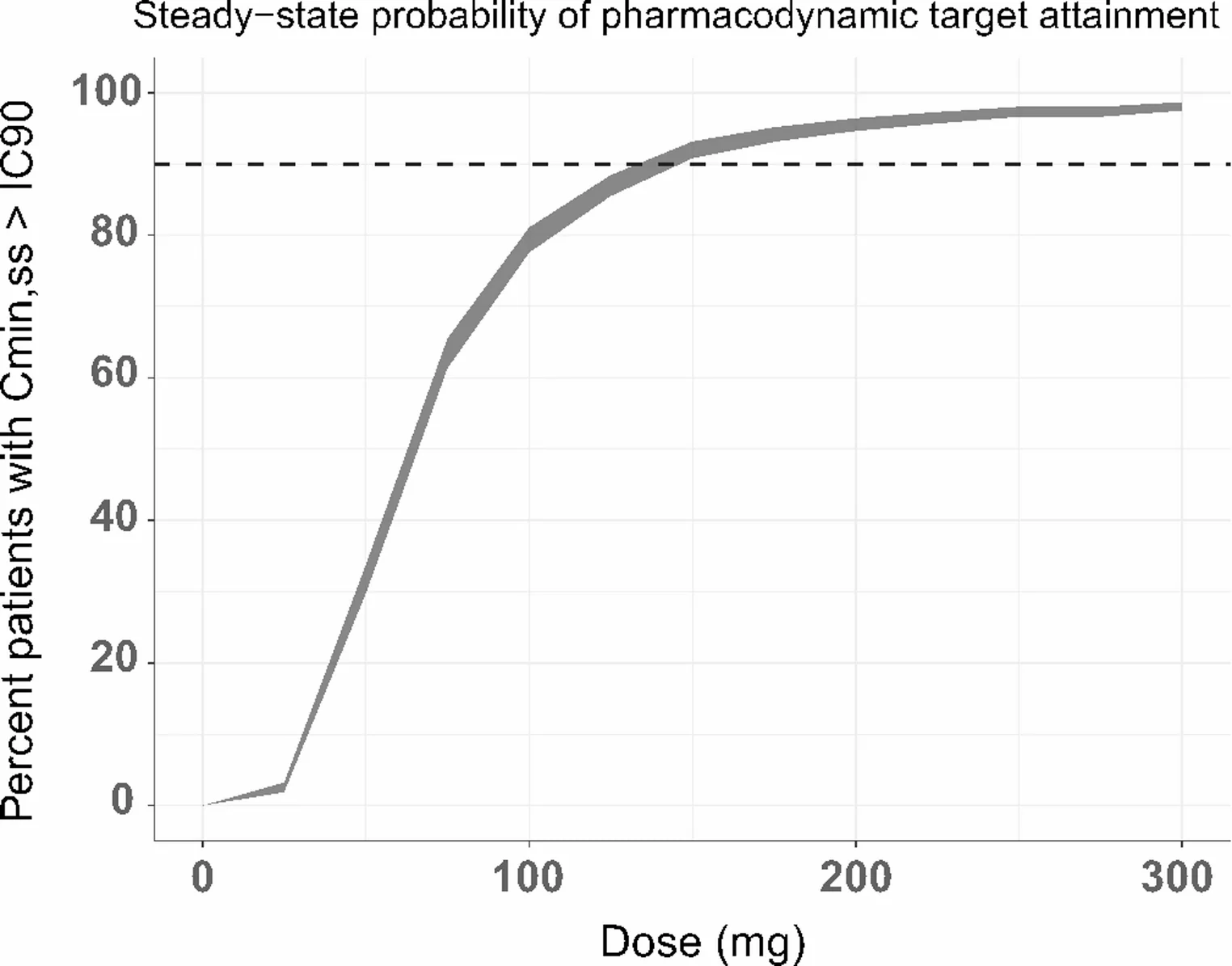
Simulated proportion of patients achieving minimum concentrations of pirtobrutinib that exceed 90% BTK inhibition. Abbreviations: Cmin = minimum concentration during a dosing interval at steady-state, IC90 = drug concentration that produces 90% of maximum inhibitory effect (Imax). Dotted line represents 90% of patients, and the shaded areas correspond to the 95% confidence intervals

These simulations demonstrate that at doses ≥ 100 mg QD, ≥ 79% or more of patients are predicted to achieve concentrations of pirtobrutinib that exceed 90% inhibition of BTK across the entire dosing interval. At the RP2D of 200 mg QD, 96% of patients are predicted to exceed 90% inhibition of BTK across the entire dosing interval (Fig. 3).

## Discussion

This study provides a comprehensive popPK analysis of pirtobrutinib, a highly selective and reversible BTK inhibitor, in patients with hematological malignancies. The findings from this analysis offer valuable insights into the factors influencing the disposition of pirtobrutinib and inform potential dose adjustments based on intrinsic factors.

The popPK model characterized pirtobrutinib PK using a 2-compartment distribution model with linear clearance and 4 transit compartments for absorption. The mean elimination half-life of approximately 18.8 h supports the once daily dosing regimen, ensuring sustained BTK inhibition throughout the dosing interval.

The analysis identified body weight, renal function, and serum albumin as statistically significant covariates affecting pirtobrutinib clearance. However, simulations suggested that the effect of body weight, renal function, and serum albumin across the range of observed values in the BRUIN Study on PK of pirtobrutinib was within the range of typical PK variability and is therefore not considered clinically meaningful (Section “Impact of covariates on pirtobrutinib PK”). Mild hepatic impairment did not significantly affect pirtobrutinib clearance, despite the metabolic profile of pirtobrutinib which involves degradation by hepatic enzymes. Due to the limited number of patients with moderate and severe hepatic impairment, and insufficient data collection in the BRUIN trial to classify patients as per Child-Pugh criteria, the impact of hepatic impairment on pirtobrutinib clearance could not be reliably concluded. A subsequent clinical pharmacology study in patients with mild, moderate and severe hepatic impairment (based on Child-Pugh) established no clinically significant differences in the PK of pirtobrutinib for any degree of hepatic impairment [15].

The high oral bioavailability and extended half-life of pirtobrutinib facilitate continuous BTK inhibition, which is crucial for its therapeutic efficacy in hematological malignancies. The recommended starting dose of 200 mg QD provides extensive and sustained BTK inhibition, with 96% of patients predicted to achieve over 90% BTK inhibition throughout the dosing interval. This level of target engagement is associated with meaningful clinical benefits and supports the use of pirtobrutinib in patients with pretreated MCL, CLL/SLL, and other B cell malignancies.

The findings from this popPK analysis have important clinical implications. Although body weight, renal function, and serum albumin were identified in the model as statistically significant covariates affecting pirtobrutinib PK, they are not considered clinically meaningful since impact on PK of pirtobrutinib was within the range of typical PK variability. Mild and moderate renal impairment were associated with changes in pirtobrutinib exposure that were within the range of typical interindividual PK variability and were not considered clinically meaningful. However, only 4 patients with severe renal impairment were included in this analysis, thus the effect of severe renal impairment could not be reliably characterized in this population PK analysis. Additionally, the lack of a significant impact of mild hepatic impairment on pirtobrutinib clearance suggests that standard dosing can be maintained in these patients, simplifying treatment protocols.

Race and ethnicity were not identified as significant covariates. Non-White participants were varied in the analysis population (Black or African American *n* = 17; Asian *n* = 39; Hispanic *n* = 23; Table 2). The Asian subgroup was of sufficient size to assess for a clinically meaningful effect on apparent clearance, and none was observed. For the smaller Black or African American and Hispanic subgroups, the ability to detect modest effects was more limited. Overall, no clinically relevant effect of race or ethnicity on pirtobrutinib disposition was identified, although definitive conclusions for the least-represented subgroups (Black or African American and Hispanic) remain constrained by sample size.

Pirtobrutinib is eliminated primarily by hepatic metabolism, mediated by CYP3A4 together with UGT1A8 and UGT1A9. In clinical drug–drug interaction studies, coadministration with itraconazole, a strong CYP3A4 inhibitor, increased pirtobrutinib AUC by 1.49-fold, whereas rifampin, a strong CYP3A4 inducer, decreased AUC by 71% [16]. These findings indicate that CYP3A4 modulation can affect pirtobrutinib exposure, particularly with induction. However, because systemic clearance is distributed across CYP3A4 and non-CYP3A pathways rather than dependent on a single dominant polymorphic enzyme, variability in CYP/UGT activity alone is unlikely to fully explain the observed interpatient variability. Consistent with this, the final popPK model estimated moderate interpatient variability in apparent clearance of 37.9% CV, while absorption-related variability was also present, with mean transit time interoccasion variability of 45.9%. Therefore, the observed variability in PK likely reflects combined contributions from clearance pathways, absorption variability, and other patient- or treatment-related factors not fully captured by the evaluated covariates.

The present analysis was conducted in patients from the BRUIN trial, and a formal comparison between healthy volunteers and patients was outside the scope of this work. Nevertheless, predicted steady-state exposure in patients receiving pirtobrutinib 200 mg QD (Cmax 6460 ng/mL; AUC₀₋₂₄ 91,300 ng·h/mL) was generally consistent with exposure predicted from healthy volunteer data (Cmax 6927 ng/mL; AUC₀₋₂₄ 109,965 ng·h/mL) reported by Tian et al. (2025) [16]. These findings do not suggest a clinically meaningful difference in overall pirtobrutinib exposure between healthy volunteers and patients with B-cell malignancies.

## Conclusion

This study provides a comprehensive characterization of pirtobrutinib PK and identifies key factors influencing its disposition. The recommended dose of 200 mg QD offers substantial BTK inhibition hence significant clinical benefits. These findings support the continued development and clinical application of pirtobrutinib in this patient population.

## Supplementary Information

Below is the link to the electronic supplementary material.

Supplementary Material 1

## Data Availability

A data sharing statement provided by the authors is available with this article. Eli Lilly and Company provides access to all individual participant data collected during the trial, after anonymization, except PK or genetic data. Data are available to request 6 months after the indication studied has been approved in the United States and European Union and after primary publication acceptance, whichever is later. Once the data is available, there is currently no set expiration date for data requests. Access is provided after a proposal has been approved by an independent review committee identified for this purpose and after receipt of a signed data sharing agreement. Data and documents, including the study protocol, statistical analysis plan, clinical study report, and blank or annotated case report forms, will be provided in a secure data sharing environment. For details on submitting a request, see the instructions provided at [www.vivli.org](https:/www.vivli.org) .
