# Supplementary material for: Population pharmacokinetic analysis of pirtobrutinib, a non-covalent BTK inhibitor, in patients with hematological malignancies from the Phase 1/2 BRUIN study
Source: Cancer Chemother Pharmacol. 2026 Sep 24;96(1):111. doi: 10.1007/s00280-026-04951-4 (PMC13612640; doi:10.1007/s00280-026-04951-4)
Supplement: Supplementary file 1 [file 280_2026_4951_MOESM1_ESM.pdf]

Supplementary Appendix

Supplement to Bell R, O'Brien LM and Eunice Yuen E et al. Population Pharmacokinetic Analysis of Pirtobrutinib, a non-covalent BTK inhibitor, in Patients with Hematological Malignancies from the Phase 1/2 BRUIN Study

Contents

Supplemental Figures 3

Figure S1. Impact of Various Factors on Pirtobrutinib Clearance 3

Figure S2. Forest plot of steady-state pirtobrutinib exposure 4

Supplemental Figures

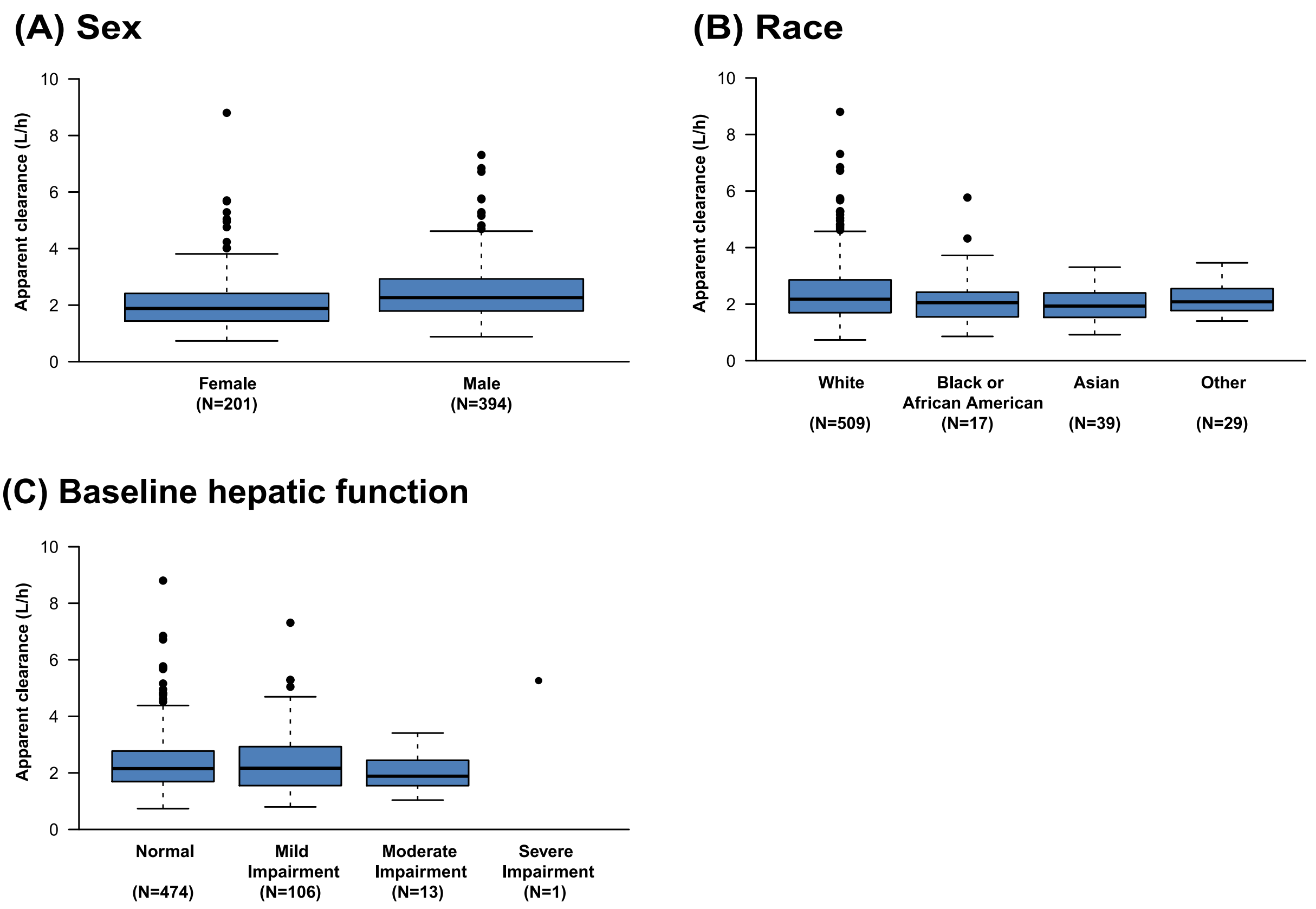

Figure S1. Impact of Various Factors on Pirtobrutinib Clearance. Box plots of apparent clearance (L/h) stratified by **(A)** sex, **(B)** race, **(C)** baseline hepatic function. NOTE: Box plots depict the 25th, 50th, and 75th percentiles. Whiskers represent 1.5 times the interquartile range.

Other races include Native Hawaiian or other Pacific Islander (n=2), American Indian or Alaska Native (n=3), and Mixed race or multiple races (n=24). Hepatic function was categorized as Normal (TBI ≤ ULN and AST ≤ ULN), Mild (TBI ≤ 1.5 • ULN and AST > ULN or ULN < TBI ≤ 1.5 • ULN), Moderate (1.5 • ULN < TBI ≤ 3 • ULN), and Severe (TBI > 3 • ULN). Race and hepatic function classification data missing for 1 patient each.

Abbreviations: AST = aspartate transaminase; eGFR, estimated glomerular filtration rate; PK, pharmacokinetic; QD, once daily; TBI, total bilirubin; ULN, upper limit of normal.

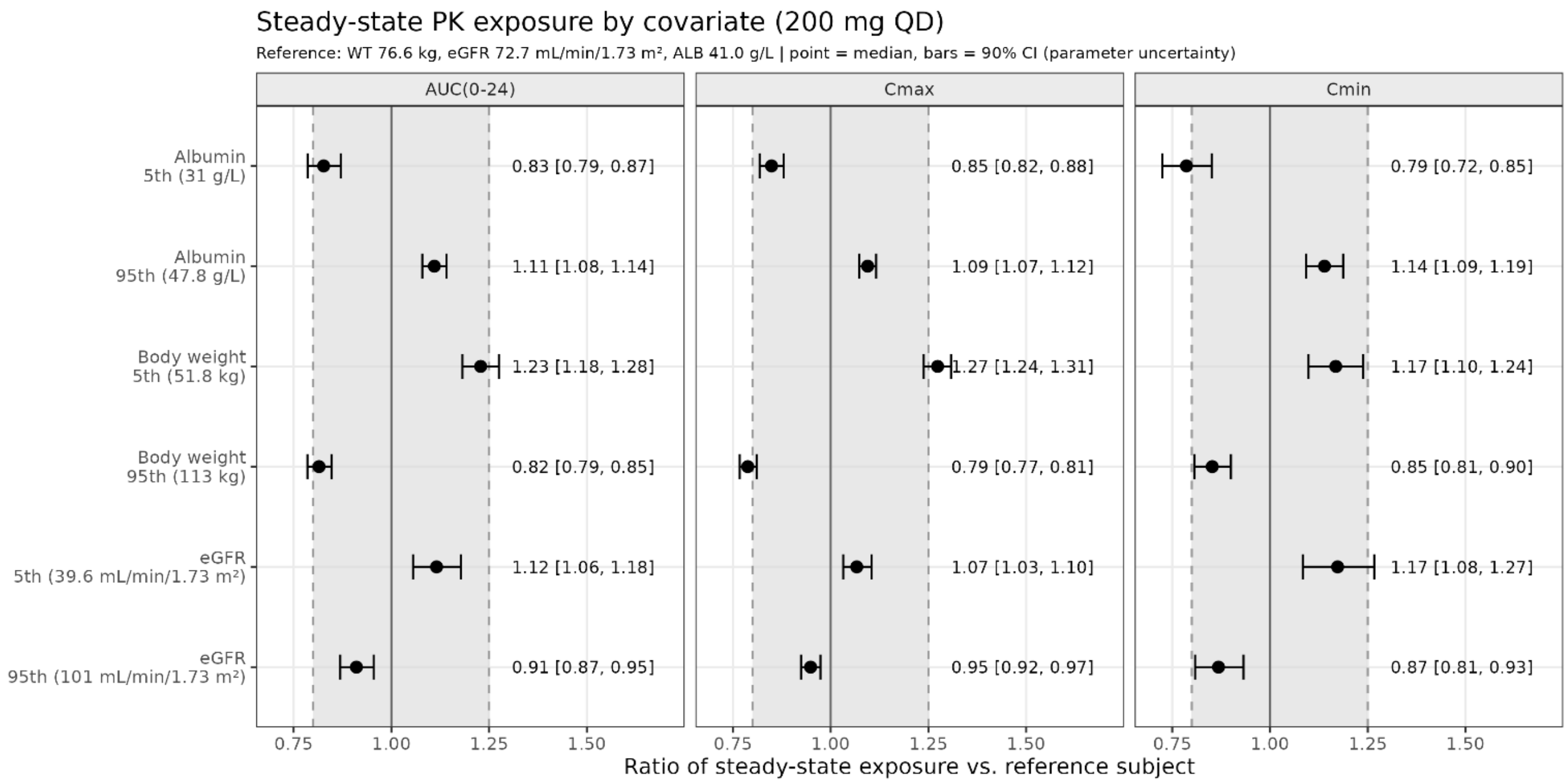

Figure S2. Forest plot of steady-state pirtobrutinib exposure ($AUC_{0–24}$, Cmax, Cmin) at the 5th and 95th percentiles of body weight, serum albumin, and eGFR at 200 mg QD, expressed as the ratio to the reference (median) patient (body weight 76.6 kg, eGFR 72.7 mL/min/1.73 m², serum albumin 41.0 g/L)
